# Low Rates of Lung and Colorectal Cancer Screening Uptake Among a Safety-net Emergency Department Population

**DOI:** 10.5811/westjem.2022.5.55351

**Published:** 2022-08-11

**Authors:** Nicholas Pettit, DuyKhanh Ceppa, Patrick Monahan

**Affiliations:** Indiana University School of Medicine, Department of Emergency Medicine, Indianapolis, Indiana

## Abstract

**Introduction:**

A suspected diagnosis of cancer through an emergency department (ED) visit is associated with poor clinical outcomes. The purpose of this study was to explore the rate at which ED patients attend cancer screenings for lung, colorectal (CRC), and breast cancers based on national guidelines set forth by the United States Preventive Services Task Force (USPSTF).

**Methods:**

This was a prospective cohort study. Patients were randomly approached in the Eskenazi Hospital ED between August 2019–February 2020 and were surveyed to determine whether they would be eligible and had attended lung, CRC, and breast cancer screenings, as well as their awareness of lung cancer screening with low-dose computed tomography (LDCT). Patients who were English-speaking and ≥18 years old, and who were not critically ill or intoxicated or being seen for acute decompensated psychiatric illness were offered enrollment. Enrolled subjects were surveyed to determine eligibility for lung, colorectal, and breast cancer screenings based on guidelines set by the USPSTF. No cancer screenings were actually done during the ED visit.

**Results:**

A total of 500 patients were enrolled in this study. More participants were female (54.4%), and a majority were Black (53.0%). Most participants had both insurance (80.2%) and access to primary care (62.8%). Among the entire cohort, 63.0% identified as smokers, and 62.2% (140/225) of the 50- to 80-year-old participants qualified for lung cancer screening. No patients were screened for lung cancer in this cohort (0/225). Only 0.6% (3/500) were aware that LDCT was the preferred method for screening. Based on pack years, 35.5% (32/90) of the patients who were 40–49 years old and 6.7% (6/90) of those 30–39 years old would eventually qualify for screening. Regarding CRC screening, 43.6% (218/500) of the entire cohort was eligible. However, of those patients only 54% (118/218) had been screened. Comparatively, 77.7% (87/112) of the eligible females had been screened for breast cancer, but only 54.5% (61/112) had been screened in the prior two years.

**Conclusion:**

Many ED patients are not screened for lung/colorectal/breast cancers even though many are eligible and have reported access to primary care. This study demonstrates an opportunity and a need to address cancer screening in the ED.

## INTRODUCTION

Obtaining a diagnosis of cancer through an emergency department (ED) visit is associated with poor outcomes and health disparities, and results in worse outcomes when compared to cancer diagnoses that are obtained through scheduled screenings.^1^ Various administrative database studies have demonstrated that diagnosing cancer through an emergent presentation is a common occurrence for ED clinicians, particularly in rural and urban EDs. While 20–50% of cancer diagnoses are made during an ED visit, little research has focused on strategies to encourage early screening to decrease the number of emergency presentations of cancer.^1^ Improved cancer screening rates across all racial and ethnic groups reduces the stage at diagnosis, with an earlier stage of diagnosis being associated with improved outcomes.^2^, ^3^

The United States Preventive Services Task Force (USPSTF) has published screening guidelines for common cancers.^4^ For lung cancer, annual low-dose computed tomography (LDCT) is recommended for adults 50–80 years old, with a smoking history within the prior 15 years of at least 20-pack years.^4^, ^5^ These guidelines were recently from the previous screening recommendation of those 55–80 years old with a 30-pack year smoking history. Annual lung cancer screening with LDCT has demonstrated a reduction in lung cancer-specific mortality of 20.0%.^6^ To detect colorectal cancer the USPSTF recommends screening between ages 50–75 with either direct visualization or a fecal immunochemical test (FIT). Lastly, the USPSTF recommends biennial screening mammography for women between the ages of 50–74. It has been reported that ED patients are disproportionately non-adherent with the USPSTF cancer screening recommendations.^7^

Disparities in cancer screening, including socioeconomic status (SES) and racial/ethnic status are pervasive in the literature, and has been further exacerbated by the coronavirus 2019 pandemic.^8^ Despite the fact that EDs serve as a safety net for vulnerable populations who suffer from health disparities, limited work has explored the missed opportunities for cancer screening among ED patients. The adherence to the recommended USPSTF cancer screening guidelines among ED populations is not known. Herein, we attempt to determine what percentage of our safety-net ED population (a lower socioeconomic, racially diverse, urban population, which we define as vulnerable) were screened for lung, colorectal, and breast cancers prior to coming to the ED, based on the USPSTF cancer screening recommendations. Knowing rates of screening for outpatients can inform ED interventions, such as cancer screening in the ED or cancer screening education.

## METHODS

This study was an observational cohort analysis, performed at Eskenazi Hospital, an inner-city, Level I trauma center/academic hospital in downtown Indianapolis. The population is racially diverse (44.5% Black) and of low SES (low income and primarily insured by Medicare/Medicaid/self-pay). The study was performed from August 2019–February 2020, under exempt status from the Indiana School of Medicine Institutional Review Board (IRB protocol #1909893946). Patients were approached and, following verbal consent to participate, were asked a series of up to 20 questions (fewer if not female, or non-smokers) to determine the patient’s adherence to known cancer screening guidelines. We obtained demographic information in addition to cancer screening questions. Previous CT was confirmed in the electronic health record. Patients were enrolled between the hours of 7 am – 3 am. Exclusion criteria were as follows:: age <18 years; non-English speaking; prisoner status; pregnant status; decompensated psychiatric illness; and critically ill or hospice status.

**Population Health Research Capsule**

What do we already know about this issue?

*Emergency department patients are thought to be less adherent to national cancer screening guidelines. However data are limited.*

What was the research question?

*What is the percent uptake of cancer screening for lung, colorectal, and breast cancers among a safety-net ED population.*

What was the major finding of the study?

*Many ED patients are not getting appropriate cancer screening, even though many are high risk, and have reported access to primary care.*

How does this improve population health?

*This work offers an opportunity for intervention to improve the cancer screening uptake among an at-risk ED population.*

Sample size was determined to provide 80% power, alpha = 0.05, using a test of sample proportion compared to a known population, based upon national averages for known screening modalities. For lung cancer, 6.1% was used as the national average for 2017, and <1% was estimated for our study population, requiring a sample size of 60 eligible for cancer screening.^9^ For colorectal cancer, 63% was used for the average, and we estimated an expected rate of 50%, needing a sample size of 111.^10^ Lastly, for breast cancer, the national average is 71.6% and we estimated 50% of our population would be screened, needing a sample size of 36.^11^ It took 500 active enrolled patients to meet the total number of eligible patients for each individual cancer screening based on age because we included patients >18 years old. A random number generator was used to identify random beds in the ED for our research staff to approach the patients.

### Outcomes

The primary outcome was the rate at which patients are screened for lung, colorectal, and breast cancers in accordance with the USPSTF cancer screening recommendations. Secondary outcomes included comparison between age groups for the lung cancer screening cohort (<30 years old, 30–39 years old, 40–49 years old, 50–80 years old, and 80+ years old), awareness that LDCT is the preferred method for lung cancer screening, and frequency at which patients will eventually qualify for lung cancer screening once they come of age.

### Analysis

We used Microsoft Excel statistical package (Microsoft Corp., Redmond, WA) for descriptive statistics. The 95% normal-approximated confidence intervals (CI) were calculated surrounding the sample proportions. A z- test comparing the single sample proportion for patients screened was compared to the above-mentioned known published population proportions.

## RESULTS

Table 1 demonstrates the descriptive analysis of the examined cohort, divided into age groups. In total, 639 patients were approached, nine declined participation, and 130 were excluded. The median age was 46.6 years old, 54% were female, and 53% Black, 42.8% White, and 7.4% Hispanic/Latino; 62.8% reported having access to a primary care physician, and 80.2% had an active insurance provider. The younger cohorts had lower rates of access to primary care and insurance.

### Adherence to Screening

Table 2 presents the adherence and outcome data for the lung cancer screening questions. We found that 63% (315/500) of participants were current smokers, with 73.8% (166/225) of 50–80-year-olds reporting smoking within the prior 15 years. Additionally, the average pack years for tobacco use for the entire cohort was 17.1 pack years; among 50–80-year-olds the average tobacco use was 28.2 pack years. Of the entire cohort, 36.6% (183/500) qualified for lung cancer screening based on the number of pack years; 62.2% (140/225) of 50–80-year-olds were eligible for lung cancer screening. Of those eligible for LDCT, no patients had been screened with a LDCT, with the one patient who had LDCT completed being under 50 years old. Meanwhile, 35.5% (32/90) of 40–49-year-olds and 6.7% (6/90) 30–39-year-olds would qualify for LDCT once they come of age, based on the USPSTF guidelines. Only 0.6% (3/500) of the entire population correctly identified that LDCT is the preferred method for lung cancer screening, despite 9.2% (46/500) stating that they knew lung cancer screening exists. Lastly, a proportion test was used to compare the one person who had been screened, albeit incorrectly, for lung cancer to the previously recorded rate of 6.1%, which resulted in a significant *P*-value of <0.001 (95% CI: <0.1% – 2.2%).

Table 3 presents the screening attendance for colorectal and breast cancers, respectively. Focusing on colorectal cancer, the number of patients who would meet screening criteria by age (ie, 50–75 years old) was 43.6% (218/500). These patients reported having high rates of primary care access and insurance, 77.5% and 91.3%, respectively. However, of those eligible for CRC screening, only 54.1% (118/218) had been screened for colorectal cancer, compared to a national average of 63% (*P* = 0.008; 95% CI: 47.3%–61.0%). Similar rates of screening attendance were observed for both Black and White patients with 54.7% of eligible White patients having been screened, compared to 51.3% of Black patients screened.

Lastly, focusing on breast cancer screening, of the 270 patients in the study, 112 met the age criteria from the USPSTF guidelines (50–74 years old). In this study 77.7% of the eligible had undergone a mammogram, which was not statistically different from the published rate of 71.6% (*P* = 0.17; 95% CI: 68.8%–85.0%). However, removing those females who last had a mammogram longer than two years prior only 54.5% (61/112, *P* <0.0001, 0.45–0.63) of eligible females were screened, which is significantly different from the published rate. The recommendation is biennial screening mammography, and the minimum/median/maximum years since the last mammogram were <0 years, 1 year, and 28 years, respectively. Thus, 77.7% is artificially higher than the likely observed adherence to the national guidelines. Again, there was a high rate of reported primary care access (89%) among this female cohort; however, there was a wide range of when females had last been screened. The observed rate of breast cancer screening in this cohort was not statistically different from the published rate of 71.6%. Lastly, 59.0% of Black patients had been screened for breast cancer in the prior two years, compared to 37.8% of White patients having been screened.

## DISCUSSION

In this study conducted within a safety-net ED healthcare system, we sought to determine the rate at which patients are screened for three of the most common, treatable, and detectable cancers based on the USPSTF cancer screening recommendations. We identified that lung cancer is uncommonly screened for despite a large portion of our ED patient population having access to primary care. There is a well-recognized predilection for heavy smoking among low SES patients, and this study adds to the body of literature suggesting a need for increased awareness of lung cancer screening within this population.^12^ Socioeconomic status, racial, and ethnic inequities among cancer screening are well established in the existing literature, and with a growing need to reduce health disparities, there is a demand to create interventions to improve cancer care for this patient population.^13^ Emergency department utilization is high among low SES populations, and thus the ED serves as a unique venue for targeting cancer screening interventions. Additionally, we included all adult patients in this study to get a sense of the rate of patients who would be eligible for lung cancer screening by pack years due to the known high rates of tobacco abuse.

Although more prevalent than lung cancer screening, colorectal cancer screening in this population still fell below the national average, at 54%. We did not differentiate between FIT tests and direct visualization, although it is likely that within this group some were overdue for their screenings. Again, while the USPSTF guidelines are well established, they don’t account for demographics or SES. Patients of lower SES and racial minorities have known lower adherence to screening guidelines, which is the patient population served by our ED.^14^ Cancer screening guidelines continually get updated, such as the recent USPSTF changes to lung cancer screening. The USPSTF very recently amended the colorectal cancer guidelines to include persons 45–49 years old, which would only further increase the number of ED patients eligible for CRC screening.^15^

Lastly, breast cancer screening appears to be the most adhered to in this study but was still below the national rate. This is likely due to more public knowledge and awareness of breast cancer and breast cancer screening. For example, national mammography screening programs and awareness interventions have led to increased self-examinations and increased likelihood of attending breast cancer screening.^16^ Comparatively, a physician usually cannot palpate an undiagnosed lung cancer as they would a breast mass; however, increasing awareness of screening and risk factors for lung cancer has been demonstrated with public campaigns in the United Kingdom, which could easily be replicated in US EDs.^17^

Lengths of stay (LOS) in the ED vary, with anecdotal examples demonstrating >6 hours for even benign problems such as wrist factures.^18^ Most of the patient time in the ED is spent waiting for laboratory/radiology testing, consultation, or even for the discharge process, leaving a large amount of time where additional services or interventions could be provided to the patient.^19^ This study demonstrates an unmet need for increasing access to cancer screening and prevention among our safety-net ED population. Increasing evidence has demonstrated that the ED is in a unique position to address disparities in cancer prevention and screening.^7^ As demonstrated in this work, ED patients are disproportionally non-adherent with the USPSTF cancer screening recommendations and, thus, the ED is a desirable location to reach these populations that otherwise would not have access to preventive services.^20^ Examples of success in addressing cancer prevention/screening in the ED include a randomized controlled trial by Adler et al.^21^ Their study demonstrated the feasibility and efficacy of a behavioral intervention to increase uptake of cervical cancer screening among ED patients at an urban, academic ED.^21^

Within our study, we observed high rates of tobacco abuse. The ED may be a suitable opportunity to intervene on these issues, which could have impacts on not only reducing cancer, but other health issues associated with obesity and tobacco use. For example, ED-initiated tobacco control has been effective in promoting continual tobacco-use abstinence up to 12 months, as demonstrated by a 2017 systematic review and meta-analysis.^22^ Additional interventions can be proposed such as ED-based screening and referral to known cancer screening programs (eg, a lung cancer screening clinic), or even cancer screening education.^23^ This concept would be to use the ED space and ED visit for comprehensive care, expanding the role that EDs could play, especially among those suffering from health disparities. Similar ideas in using the ED space for care beyond emergencies has been demonstrated for other chronic and treatable medical conditions, such as ED-based HIV screening fir human immunodeficiency virus.^20^ To accomplish this, resource-neutral interventions must be developed, as to not overburden ED clinicians, who in many instances are already resource limited. Peer recovery coaches are frequently used in the ED to address opioid use disorder; similar coaches or patient advocates could be used to discuss cancer screening and prevention with patients, thereby not overburdening ED clinicians.^24^ While we should focus on reducing ED crowding and LOS, especially for minor complaints, the ED visit may represent the only time that many uninsured and underserved patients access healthcare.^25^

## LIMITATIONS

Our results likely overestimate the percentage of patients screened as we eliminated non-English speaking patients. Eskenazi serves a large Latin-X, Spanish-speaking, population, many of whom use the ED as their primary source of medical care. This was a convenience sample; however, patients were enrolled between the hours of 7 am–3 am, and the overall demographics are similar to the general population seen in the Eskenazi ED. Additionally, we included 500 patients in this study to reach the required number of patients who would meet the screening guidelines, which included many younger individuals who do not usually need to be aware of CRC or lung cancer screening for some time. To that end though, this gives us some insight into the social risks including tobacco use among this patient population, as well as obesity, which affords opportunities for public health interventions. Lastly, recall bias is a possible limitation in that interviewers were relying on patient recollection for actual cancer screening. Overall, we believe this study is likely generalizable to other inner-city, county hospitals, as most county hospitals serve a similar population; however, these results are likely not generalizable outside of that context.

## CONCLUSION

This study demonstrates that among a random sample of ED patients with a high rate of tobacco use, there are poor rates of cancer screening attendance for lung, colorectal, and breast cancers. Additionally, our safety-net ED population demonstrates cancer screening attendance rates lower than the national average. Earlier detection of asymptomatic malignancies is associated with higher likelihood of survival and cure rates; thus, this work provides the framework for novel ED-based interventions using a patient’s ED visit as a window of opportunity for intervention.^2^ Furthermore, many younger ED patients (ages 30–49) would qualify for lung cancer screening based on their tobacco use. Knowledge of the rates of cancer screening attendance for outpatients who frequent the ED can guide ED interventions, such as ED cancer screening or cancer screening education.

## Figures and Tables

**Table 1:** Demographics and characteristics for the study population.

Demographics	Overall, N = 500 (total number)	95% CI for sample proportion for entire population	<30 years old (n = 89)	30–39 years old (n = 90)	40–49 years old (n = 90)	50–80 years old (n = 225)	80+ years old (n = 6)
Median age	46.6		23.5	34	45	59	83.5
Female	54.4% (270)	0.49–0.58	59.6% (53)	50.0% (45)	56.6% (51)	51.5% (116)	83.3% (6)
Race							
Black	53.0% (265)	0.48–0.57	55.1% (49)	41.1%(46)	41.1%(46)	52.9%(46)	16.6% (1)
White	42.8% (214)	0.38–0.47	38.2% (34)	45.5% (41)	44.4% (90)	43.6% (98)	83.3% (5)
Other	7.4% (37)	0.02–0.06	12.4% (11)	11.1% (10)	7.8% (7)	4.0% (9)	0% (0)
Access to primary care physician - yes	62.8% (314)	0.33–0.41	43.8% (39)	44.4% (40)	60% (54)	77.8% (175)	100% (6)
Have insurance - yes	80.2% (401)	0.76–0.83	70.8% (63)	63.3% (57)	77.7% (70)	91.1% (205)	100% (6)

*CI*, confidence interval.

**Table 2:** Outcomes data for lung cancer screening.

	Overall, N = 500 (total number)	95% CI for sample proportion	<30 years old (n = 89)	30–39 years old (n = 90)	40–49 years old (n = 90)	50–80 years old (n = 225)	80+ years old (n = 6)
Active smoker within 15 years - yes	63.0% (315)	0.59–0.67	41.6% (37)	57.8% (90)	64.4% (58)	73.8% (166)	33.3% (2)
Average pack years	17.1		2.1	7.01	13.1	28.2	25.8
Qualify for LDCT (by pack years)	36.6% (183)		1.1% (1)	6.7% (6)	35.5% (32)	62.2% (140)	66.7% (4)
Had a CT chest	7.6% (38)	0.05–0.10	2.2% (2)	3.3% (3)	5.6% (5)	12.0% (27)	16.7% (1)
Had LDCT for lung cancer screening	0.2% (1)	<0.001–0.10	0%	0%	0.4% (1)	0%	0%
Do you know lung cancer screening exists? -yes	9.2% (46)	0.07–0.12	2.2% (2)	1.1% (1)	10% (9)	14.7% (33)	16.7% (1)
Correctly stated CT scan is preferred for screening	0.6% (3)	0.002–0.02	0%	0%	0%	1.3% (3)	0%

*CI*, confidence interval; *CT*, computed tomography; *LDCT*, low-dose computed tomography.

**Table 3:** Adherence to colorectal and lung cancer screening guidelines.

	Percent (total number)	95% CI for sample proportion
Colorectal Cancer Screening Adherence		
How many 50–75	43.6% (218)	0.39–0.48
Of 218, has primary care physician (PCP)	77.5% (169)	0.71–0.82
Has insurance	91.3% (199)	0.87–0.95
Of 218, screened for CRC?	54.1% (118)	0.47–0.61
Frequency of White patients screened	54.7% (52/95)	0.47–0.65
Frequency of Black patients screened	51.3% (60/117)	0.42–0.63
Breast Cancer Screening Adherence		
Number of females	54% (270/500)	0.50–0.58
Number of females meeting screening criteria	41.5% (112/270)	0.36–0.48
Have PCP	89% (241/270)	0.85–0.93
Screened for breast cancer with mammogram	77.7% (87/112)	0.69–0.85
Screened for breast cancer with mammogram within last 2 years	54.5% (61/112)	0.45–0.63
Number of years since last mammogram (min/median/max)	0, 1, 28 years	
Frequency of white patients screened within last 2 years	37.8% (17/45)	0.24–0.52
Frequency of black patients screened within last 2 years	59.0% (36/61)	0.47–0.71

*CI*, confidence interval; *PCP*, primary care physician; *CRC*, colorectal cancer.
